# Mechanism for the Increased Permeability in Endothelial Monolayers Induced by Elastase

**DOI:** 10.1155/S0962935194000025

**Published:** 1994

**Authors:** N. Suzuki, Y. Ishii, S. Kitamura

**Affiliations:** Department of Pulmonary Medicine Jichi Medical School Tochigi Minamikawachi 329-04 Japan

## Abstract

The aim of this study was to investigate the mechanism for the increase in endothelial permeability induced by human neutrophil elastase (HNE). Pretreatment of bovine pulmonary artery endothelial cells (BPAEC) with HNE(0-30 *μ*g/ml) for 1 h produced a concentration dependent increase in ^125^I-albumin clearance. The effect was reversible and was not due to cytolysis. Pretreatment of BPAEC with sodium tungstate, which depletes xanthine oxidase, or with oxypurinol, did not prevent HNE induced increased permeability. Heparin, which neutralizes the cationic charge of HNE, also had no protective effect. Pretreatment with heat inactivated HNE, which still had positive charge sites, did not result in increased endothelial permeability. Also, ONO-5046, a novel specific inhibitor of HNE, did prevent increased permeability. These results suggest that elastase increases endothelial permeability mainly through its proteolytic effects.


Mediators of Inflammation 3, 11–16 (1994)

### Mechanism for the increased permeability in endothelial monolayers induced by elastase

N. Suzuki[CA], Y. Ishii and S. Kitamura

Department of Pulmonary Medicine, Jichi Medical School, Minamikawachi, Tochigi, 329-04, Japan

[CA] Corresponding Author

**THE aim of this study was to investigate the mechanism for the increase in endothelial permeability induced by human neutrophil elastase (HNE). Pretreatment of bovine pulmonary artery endothelial cells (BPAEC) with HNE(0–30 μg/ml) for 1 h produced a concentration dependent increase in $^{125}$I-albumin clearance. The effect was reversible and was not due to cytolysis. Pretreatment of BPAEC with sodium tungstate, which depletes xanthine oxidase, or with oxypurinol, did not prevent HNE induced increased permeability. Heparin, which neutralizes the cationic charge of HNE, also had no protective effect. Pretreatment with heat inactivated HNE, which still had positive charge sites, did not result in increased endothelial permeability. Also, ONO-5046, a novel specific inhibitor of HNE, did prevent increased permeability. These results suggest that elastase increases endothelial permeability mainly through its proteolytic effects.**

**Key words:** Bovine pulmonary artery endothelial cells, Cationic charge, Human neutrophil elastase, Permeability, Proteolytic activity, Xanthine oxidase

### Introduction

Polymorphonuculear leukocytes (PMN) contribute to the development of high-permeability lung oedema. Several studies in models of acute lung injury indicate that PMN adhere to the microvascular endothelium, extravasate and then accumulate.[1–4] Bronchoalveolar lavage fluid of patients with adult respiratory distress syndrome shows an increase in PMN.[5,6] Furthermore, rendering animals neutropenic or administering monoclonal antibodies against neutrophil adhesion molecules affords significant protection against models of the adult respiratory distress syndrome.[7–9] About 3–4 μg of elastase is found in $10^6$ PMN,[10,11] and these cells release 20–30% of their elastase content[12] when they are activated and adhere to endothelial cells.[13] The exposure of endothelial cells to elastase increases the endothelial permeability.[14–16] The mechanism of the elastase induced increase in permeability has not been fully elucidated.

Rodell *et al.*[14] reported that xanthine oxidase (XO) mediates elastase induced endothelial injury in models of isolated lungs and endothelium. Peterson *et al.*[15] demonstrated that the positive charge of elastase is most important for increased transendothelial albumin transfer. Prolonged exposure to PMN elastase releases $^{51}$Cr from prelabelled endothelial cells,[17,18] suggesting cytolysis as a cause of hyperpermeability. Because PMN elastase degrades the extracellular matrix proteins such as collagen and fibronectin,[19] the proteolytic ability of elastase may play an major role in the increased permeability of endothelial cells by altering the integrity of the extracellular matrix.

In the present study the effects of elastase on monolayers of bovine pulmonary artery endothelial cells (BPAEC) were examined and the mechanism of the elastase induced increase in endothelial permeability was investigated. The roles of the positive charge, XO activity, and the enzymatic proteolytic effect of elastase were also examined.

### Materials and Methods

*Reagents:* Human neutrophil elastase (HNE) was purchased from Elastin Products Co., Inc. (Owensville, MO, USA). It was prepared from sputum obtained from patients with cystic fibrosis, affinity purified on soluble elastin covalently linked to agarose beads, and further purified by affinity chromatography on agarose. The final product is free of cathepsin G, myeloperoxidase and lysozyme. The solution containing HNE was negative for endotoxin, as determined by the Limulus lysate test.[20]

Sodium tungstate, oxypurinol, heparin, gelatin, bovine fibronectin, bovine serum albumin (fraction V) and hydroxyethyl piperazine ethane sulfonic acid (HEPES) were purchased from Sigma Chemical Co. (St Louis, MO, USA). Dulbecco's modified Eagle's medium (DMEM) and foetal bovine serum (FBS) were purchased from Gibco Laboratories (Grand Island, NY, USA), and $^{125}$I-albumin from

© 1994 Rapid Communications of Oxford Ltd

New England Nuclear (Boston, MA, USA). A new specific elastase inhibitor, ONO-5046, *N*-[2-[4-(2,2-dimethylpropionyloxy)phenylsulfonyl-amino]benzoyl] aminoacetic acid, was provided by Ono Pharmaceutical Co. (Osaka, Japan).

*Preparation of cells:* The BPAEC were freshly isolated, grown in DMEM supplemented with 10% FBS, and used between passages 18 and 22. These cells were characterized as endothelial in origin by the presence of angiotensin converting enzyme activity and factor VIII related antigen. The endothelium had typical cobblestone morphology with tight interendothelial junctions. A total of $1 \times 10^5$ BPAEC in 0.5 ml were seeded and allowed to grow to a confluent monolayer.

*Preparation of monolayers on filters:* Polycarbonate filters with 0.8 $\mu$m pore in diameter (Nucleopore; Pleasanton, CA, USA) were washed in 0.1% acetic acid and coated with gelatin as described previously,[21] and glued to the bottom of plastic cylinders that had an inside diameter of 9 mm (Adaps, Inc.; Dedham, MA, USA). The wells were suspended in 24-well culture plates, sterilized by ultraviolet light for 24 h, and then coated with bovine fibronectin, 30 $\mu$g/ml. The BPAEC were seeded onto the filters and maintained at 95% humidity, 5% $CO_2$, 37°C. They were used 4–5 days after seeding.

*Transendothelial albumin assay:* The system for determining transendothelial $^{125}$I-albumin flux was described previously.[22] Wells containing the endothelial monolayers served as luminal chambers and were floated by means of a styrofoam collar in a large abluminal chamber. The abluminal chamber contained 25 ml of Hank's balanced salt solution (HBSS) with 20 mM HEPES and 0.5% fraction V bovine serum albumin, which was stirred continuously for complete mixing and maintained at a constant temperature of 37°C. The luminal chamber contained 650 $\mu$l of identical medium to which $^{125}$I-albumin was added as tracer. With the chamber sitting in the well, the levels of medium in the well and in the abluminal were the same, so no hydrostatic pressure was generated across the monolayer.

The buffer in the abluminal chamber was sampled every 5 min for 1 h. The $^{125}$I-activity was measured in a gamma counter (Aloka; Tokyo, Japan), and the transendothelial clearance rate ($\mu$l/min) was calculated by weighted least-squares non-linear regression (BMDP Statistical Software; Berkeley, CA, USA).[22] The clearance rate was corrected for differences in the ratio of free to bound $^{125}$I; free $^{125}$I concentrations were determined by trichloroacetic acid precipitation.

*Treatment of endothelial monolayers with HNE:* Since FBS in the culture medium contains several antiproteases,[23] BPAEC monolayers were washed twice gently with HBSS, then filled with 650 $\mu$l of HBSS containing tracer $^{125}$I-albumin and several concentrations of HNE (0, 0.1, 1, 5, 10, 30 $\mu$g/ml). After incubation for 1 h, transendothelial albumin clearance was measured. The time course of the elastase induced increase in permeability was also examined. After incubation of monolayers with HNE (1 $\mu$g/ml) for 0–6 h, transendothelial albumin clearance was measured for 1 h. In addition, the reversibility of the response was examined. The BPAEC monolayers were treated with 0, 1 or 10 $\mu$g/ml of HNE for 1 h, washed twice with HBSS, and incubated for 12 h in DMEM containing 10% FBS without HNE before transendothelial albumin permeability was measured.

*Culture of BPAEC with tungsten:* Tungsten is known to inactivate XO by blocking incorporation of molybdenum. To deplete intracellular XO from endothelial cells, the BPAEC were cultured and passed three times through DMEM containing sodium tungstate (10 ppm). After the sub-culture, these cells were seeded on the filters and the albumin clearance was measured.

*Effect of heat inactivation:* To evaluate the effect of enzymatic activity of HNE on transendothelial permeability, HNE was heated to 100°C for 30 min. The heating resulted in a 98% reduction in specific elastolytic activity, as measured by release of soluble elastin fragments from radiolabelled insoluble bovine nuchal elastin.[15]

*Effects of oxypurinol, heparin and ONO-5046:* Oxypurinol was used as an XO inhibitor. Heparin has a negative charge and was used to neutralize the positive charge of the endothelial cell surface. ONO-5046 competitively inhibits the proteolytic activity of HNE ($IC_{50}$ = 0.044 $\mu$M, $K_i$ = 0.2 $\mu$M).[24]

The BPAEC monolayers were treated with oxypurinol (500 $\mu$M), heparin (30 $\mu$g/ml) and ONO-5046 ($10^{-6}$ M), 30 min prior to addition of HNE (0, 1 or 10 $\mu$g/ml). After incubation for 1 h, transendothelial albumin clearance was measured.

*Lactate dehydrogenase assay:* Release of lactate dehydrogenase (LDH) from BPAEC was determined to assess whether the permeability increasing effects of HNE were due to cytolysis. Endothelial monolayers were plated on 24-well culture plates. When confluent, monolayers were washed twice with phosphate buffered saline and incubated with HBSS containing HNE (0, 1 or 10 $\mu$g/ml) for 1 h. The LDH activity in the culture medium was assayed using an LDH assay kit (Wako; Osaka, Japan). Released LDH was expressed as a percentage of total cellular LDH, which was determined after cell lysis with 1% Triton X-100.

*Statistical analysis:* Values are expressed as mean ± S.E.M. In the two-group experments, differences were compared by the *t*-test. Multi-group comparisons were made by one-way analysis of variance. A level of $p < 0.05$ was accepted as statistically significant.

### Results

*Effects of HNE on clearance of transendothelial albumin:* HNE increases the permeability of the endothelial monolayer in a concentration dependent manner (Fig. 1). The time course of the HNE induced increase in albumin transfer is shown in Fig. 2.

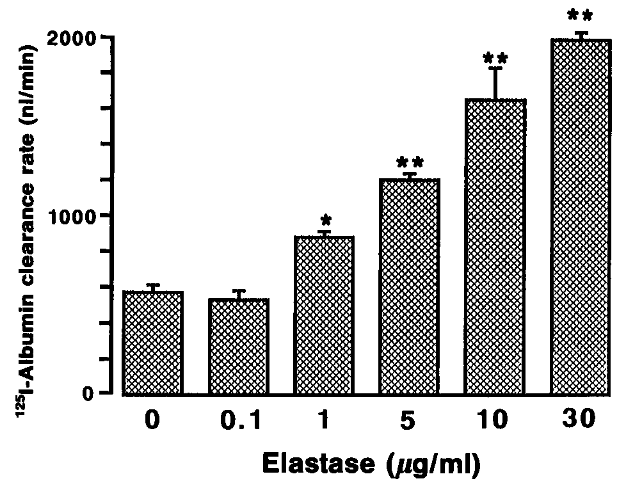

FIG. 1. Effect of human neutrophil elastase (HNE) on transendothelial $^{125}$I-albumin clearance across the bovine pulmonary vascular endothelial monolayers. Albumin clearance rates were measured after 1 h incubation with various concentrations of HNE. Values are means ± S.E.; $n = 8$ in each group, $^{*}p < 0.05$ and $^{**}p < 0.01$ compared with control.

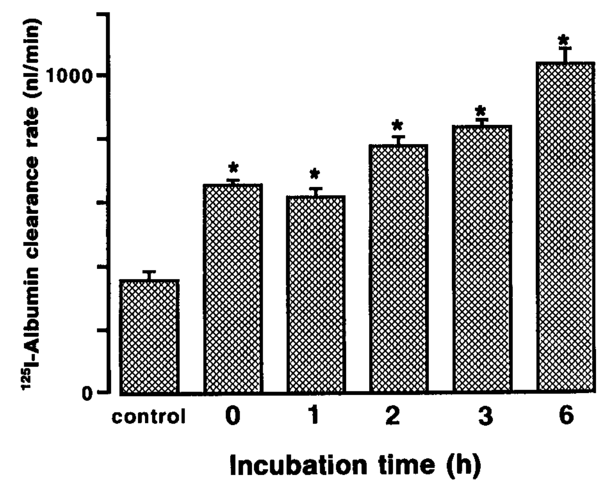

FIG. 2. Time course of the elastase induced increase in endothelial permeability. Bovine pulmonary artery endothelial monolayers were incubated with 1 μg/ml of human neutrophil elastase (HNE) for 0–6 h before transendothelial $^{125}$I-albumin clearance was measured. Values are means ± S.E.; $n = 6$ in each group. $^{*}p < 0.05$ compared with control monolayers without HNE.

**Table 1.** Permeability of BPAEC monolayers after 1 h treatment with HNE followed by reincubation in fresh medium

| Elastase (μg/ml) | $^{125}$I-Albumin clearance (μl/min) | |
|---|---|---|
| | Control | Reincubation |
| 0 | 630.8 ± 44.7 | 580.8 ± 28.0 |
| 1 | 850.2 ± 11.2 | 539.3 ± 36.9** |
| 10 | 1270.8 ± 26.5 | 455.7 ± 20.7** |

Bovine pulmonary artery endothelial cell (BPAEC) monolayers were treated with human neutrophil elastase (HNE) for 1 h and then with fresh medium for 12 h before permeabilities were determined. Values are means ± S.E.; $n = 6$ in each group. $^{**}p < 0.01$ compared with control.

Longer exposure to HNE produced a greater increase in permeability. The increased permeability induced by HNE was reversed after 12 h incubation of BPAEC with DMEM not containing HNE (Table 1).

*Effect of XO inhibition:* Pretreatment of BPAEC with oxypurinol, a potent XO inhibitor, did not prevent the HNE induced increased permeability (Fig. 3). Control and tungsten treated BPAEC had the same growth speed and the same morphological characteristics. Depletion of XO in BPAEC by tungsten treatment had no preventative effect (Fig. 4).

*Effect of positive charge:* Pretreatment of BPAEC with heparin, which neutralizes the cationic charge of HNE, did not have any protective effect on the HNE induced increase in permeability (Fig. 5). On the other hand, heat-inactivated HNE, which still had some positive chargé sites, did not produce the

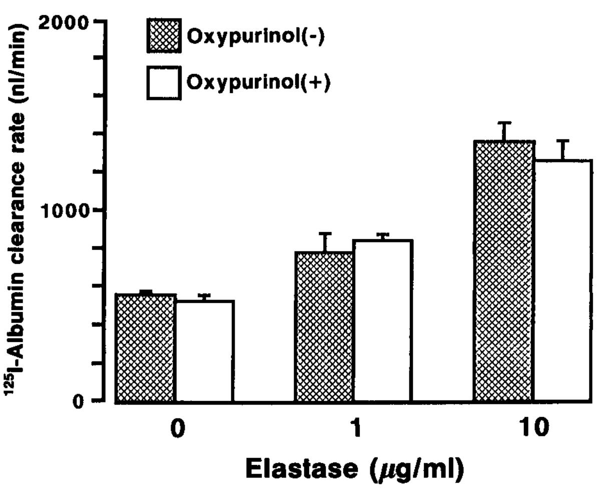

FIG. 3. Effect of oxypurinol on the human neutrophil elastase (HNE) induced increase in permeability. Bovine pulmonary artery endothelial monolayers were preincubated with (open bars) or without (cross-hatched bars) 500 μM of oxypurinol for 30 min. Transendothelial $^{125}$I-albumin clearance rates were measured without HNE and after addition of HNE (1 or 10 μg/ml) for 1 h. Values are means ± S.E.; $n = 6$ in each group.

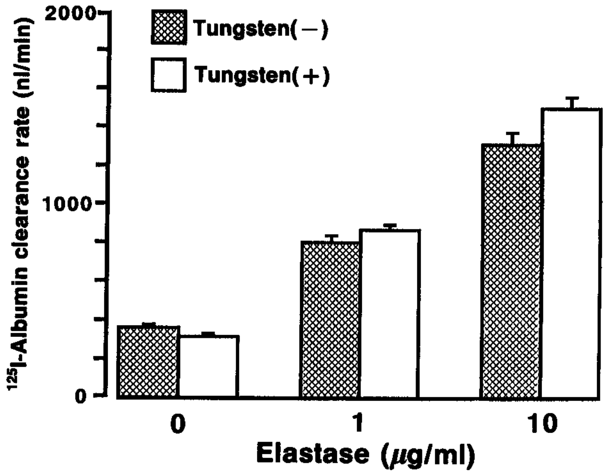

FIG. 4. Effect of tungsten treatment on the human neutrophil elastase (HNE) induced increase in permeability. Bovine pulmonary artery endothelial cells were cultured during three passages with (open bars) and without (cross-hatched bars) medium containing 10 ppm of sodium tungstate. Transendothelial $^{125}$I-albumin clearance rates were measured without HNE and after addition of HNE (1 or 10 μg/ml) for 1 h. Values are means ± S.E.; $n = 6$ in each group.

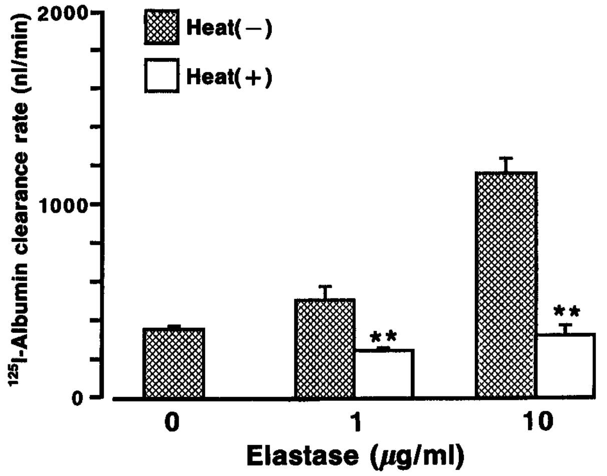

FIG. 6. Effect of human neutrophil elastase (HNE) and heat inactivated HNE on endothelial permeability. Bovine pulmonary artery endothelial monolayers were treated with HNE (cross-hatched bars) or with heat inactivated HNE (open bars) for 1 h; heat inactivation was achieved at a temperature of 100°C for 30 min. Transendothelial $^{125}$I-albumin clearance rates were measured. Values are means ± S.E.; $n = 6$ in each group. $^{**}p < 0.01$ compared with monolayers with HNE.

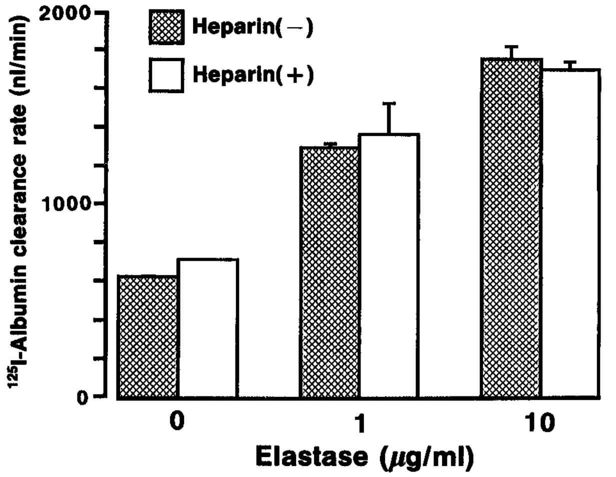

FIG. 5. Effect of heparin on the human neutrophil elastase (HNE) induced increase in permeability. Bovine pulmonary artery endothelial monolayers were preincubated with (open bars) or without (cross-hatched bars) 30 μg/ml of heparin for 30 min. Transendothelial $^{125}$I-albumin clearance rates were measured without HNE and after addition of HNE (1 or 10 μg/ml) for 1 h. Values are means ± S.E.; $n = 6$ in each group.

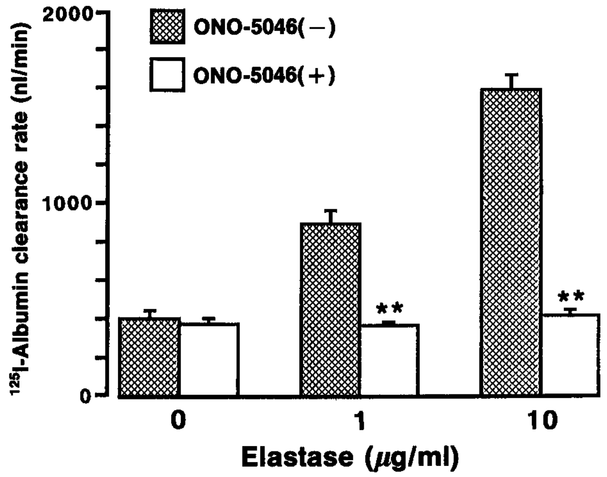

FIG. 7. Effect of ONO-5046 on the human neutrophil elastase (HNE) induced increase in permeability. Bovine pulmonary artery endothelial monolayers were preincubated with (open bars) or without (cross-hatched bars) $10^{-6}$ M of ONO-5046 for 30 min. Transendothelial $^{125}$I-albumin clearance rates were measured without HNE and after addition of HNE (1 or 10 μg/ml) for 1 h. Values are means ± S.E.; $n = 6$ in each group. $^{**}p < 0.01$ compared with monolayers with HNE.

increase in the usual transendothelial albumin transfer (Fig. 6).

*Effect of enzymatic activity:* Pretreatment of BPAEC with ONO-5046, a specific inhibitor of HNE, completely prevented the HNE induced hyperpermeability (Fig. 7). Heat inactivation of enzymatic activity of HNE also prevented the increase in permeability (Fig. 6).

*Cytotoxicity assay of elastase:* Treatment of BPAEC with elastase for 1 h did not increase LDH release (Table 2). This finding indicates that the increase in endothelial permeability was not due to a cytolytic effect of HNE.

**Table 2.** LDH release from BPAEC monolayers

| Elastase (μg/ml) | % LDH |
|---|---|
| 0 | 5.6 ± 1.2 |
| 1 | 3.4 ± 1.5 |
| 10 | 5.7 ± 2.2 |

Bovine pulmonary artery endothelial cell (BPAEC) monolayers were treated for 1 h with elastase. Values are means ± S.E.; $n = 6$ in each group. %LDH = (LDH in medium/[LDH in medium + LDH in cell lyaste]) × 100.

### Discussion

Several studies have indicated that HNE increases transendothelial permeability,[14–16] but the mechanism has not yet been fully elucidated. Tungsten is known to inactivate XO by blocking incorporation of molybdenum.[25] Rodell *et al.*[14] found that lungs isolated from rats fed a tungsten-rich diet had negligible XO activity. After exposure to hyperoxia, these lungs developed less acute oedematous injury during perfusion with HNE than XO replete lungs from control rats. In addition, tungsten treated, XO depleted cultured BPAEC made less superoxide anion, and the BPAEC monolayers leaked less $^{125}$I labelled albumin after exposure to HNE, 5 $\mu$g/ml for 3 h than did XO replete endothelial cell monolayers.[14] In the present study, however, tungsten treatment did not have any protective effect on the HNE induced increase in endothelial permeability. Superoxide anion production in the culture supernatant after exposure of endothelial cells to HNE was not significantly affected by tungsten treatment (data not shown). In addition, oxypurinol, which depletes XO activity,[26] did not prevent the HNE induced increase in albumin transfer. These data suggested that XO is not involved in the mechanism of HNE induced increase in endothelial permeability.

Endothelial cells have a negatively charged glycocalyx on their luminal surface, which may be important in controlling the escape of water and macromolecules from the vascular space.[27] Since HNE is positively charged at physiological pH,[28–30] there is a possibility that HNE increases endothelial permeability by its neutralizing effect on the negatively charged endothelial barrier. Peterson *et al.*[15] showed that heat-inactivated HNE (30 $\mu$g/ml) migrated the same distance in non-equilibrium disc gel electrophoresis as did native HNE and still had increased transendothelial albumin transfer. They also indicated that heparin neutralized the cationic charge sites on HNE and blocked the HNE induced increase in albumin transfer. In the present study, heat inactivated HNE did not increase permeability. Heparin did not prevent the HNE induced increased permeability. With HNE, concentrations of 1 and 10 $\mu$g/ml, positive charge did not have any effect on the HNE induced increase in transendothelial albumin clearance.

Heat inactivated HNE, which has specific elastolytic activity of only 2%,[15] did not increase permeability. ONO-5046, which competitively inhibits HNE,[24] completely blocked the HNE induced increase in albumin clearance. These results suggest that enzymatic activity of HNE is essential for the increase in endothelial albumin clearance.

HNE has nonspecific proteolytic activity, such that long-term exposure to this substance eventually produces cytolysis. Varani *et al.*[17] reported that HNE (5 $\mu$g/ml) caused endothelial cell killing only after prolonged incubation (longer than 8 h). Inauen *et al.*[18] reported that incubation with HNE (0.5–1.5 $\mu$g/ml) for 8 h increased detachment of endothelial cells exposed to anoxia reoxygenation but did not affect $^{51}$Cr release. They suggested that a low dose of HNE does not cause cytolysis but does cause cell dysfunction. In the present study, the exposure of BPAEC to 1 or 10 $\mu$g/ml of HNE for 1 h did not release LDH but did increase permeability, indicating that the increase in permeability was not due to cytolysis. The reversibility of the response supports this conclusion.

In the present experiment, only enzymatic inactivation prevented the HNE induced increase in transendothelial albumin clearance. This finding suggests that HNE increases endothelial permeability mainly by its enzymatic proteolytic effect.

HNE degrades a variety of important structural proteins, such as elastine, proteoglycan, collagen type I, II, III and IV, and fibronectin.[31] Weiss *et al.*[32] reported that both normal and chronic granulomatous disease neutrophils degraded the subendothelial matrix secreted by human endothelial cells by an elastase dependent process. Furthermore, Key *et al.*[33] demonstrated that HNE causes cleavage of cellular proteoglycans.

The extracellular matrix associated with vascular endothelial cells *in vivo* plays an important role in the extravasation of plasma proteins and blood cells.[33] Recently, Partridge *et al.*[34] using an endothelial monolayer treated with tumour necrosis factor $\alpha$, reported that the increase in permeability involves the loss of fibronectin and remodelling of the extracellular matrix. This report supports our hypothesis that HNE increases permeability via the proteolytic cleavage of extracellular matrix. Our study further suggests that the inhibitor of proteolytic activity of HNE might protect against elastase induced acute lung injury.

### References

1. Meyrick B. Pathology of the adult respiratory distress syndrome. *Crit Care Clin* 1986; **2**: 405–428.
2. Taylor RG, McCall CE, Thrall RS, Woodruff RD, O'Flaherty JT. Histopathological features of phorbol myristate acetate-induced lung injury. *Lab Invest* 1985; **52**: 61–70.
3. Rinaldo JE, Dauber JH, Christman J, Rogers RM. Neutrophil alveolitis following endotoxemia: enhancement by previous exposure to hyperoxia. *Am Rev Respir Dis* 1984; **130**: 1065–1071.
4. Eiermann GJ, Dickey BF, Thrall RS. Polymorphonuclear leukocyte participation in acute oleic acid induced lung injury. *Am Rev Respir Dis* 1983; **128**: 845–850.
5. Weiland JE, Davis WB, Holter JF, Mohammed JR, Dorinsky PM, Gadek JE. Lung neutrophils in the adult respiratory distress syndrome: clinical and pathophysiologic significance. *Am Rev Respir Dis* 1986; **133**: 218–225.
6. Fowler AA, Hyers TM, Fisher BJ, Bechard DE, Centor RM, Webster RO. The adult respiratory distress syndrome: cell populations and soluble mediators in the air spaces of patients at high risk. *Am Rev Respir Dis* 1987; **136**: 1225–1231.
7. Heflin AC, Brigham KL. Prevention by granulocyte depletion of increased vascular permeability of sheep following endotoxemia. *J Clin Invest* 1981; **68**: 1253–1260.

8. Flick MR, Perel A, Staub NC. Leukocytes are required for increased lung microvascular permeability after microembolization in sheep. *Circ Res* 1981; **48**: 344–351.
9. Vedder NB, Winn RK, Rice CL, Chi EY, Arfors KE, Harlan JM. A monoclonal antibody to the adherence-promoting leukocyte glycoprotein, CD 18, reduces organ injury and improves survival from hemorrhagic shock and resuscitation in rabbits. *J Clin Invest* 1988; **81**: 939–944.
10. Ohlsson K. Interactions between granulocyte proteases and protease inhibitors in the lung. *Bull Eur Physiopathol Respir* 1980; **16(Suppl)**: 209–222.
11. Weitz JI, Huang AJ, Landman SL, Nicholson SC, Silverstein SC. Elastase mediated fibrinogenolysis by chemoattractant-stimulated neutrophils occurs in the presence of physiologic concentrations of antiproteases. *J Exp Med* 1987; **166**: 1836–1850.
12. Grant L. The sticking and emigration of white blood cells in inflammation. In: Zweifach BW, Grant L, McCluskey RT, eds. *In the Inflammation Process.* New York: Academic Press, 1973; **2**: 205–249.
13. Plow EF. Leukocyte elastase release during blood coagulation. A potential mechanism of the alternative fibrinolytic pathway. *J Clin Invest* 1981; **69**: 564–572.
14. Rodell TC, Cheronis JC, Ohnemus CL, Piermattei DJ, Repine JE. Xanthine oxidase mediates elastase-induced injury to isolated lungs and endothelium. *J Appl Physiol* 1987; **63**: 2159–2163.
15. Peterson MW, Stone P, Shasby DM. Cationic neutrophil proteins increase transendothelial albumin movement. *J Appl Physiol* 1987; **62**: 1521–1539.
16. Killackey JJF, Killackey BA. Neutrophil-mediated increased permeability of microcarrier-cultured endothelial monolayers: a model for the *in vitro* study of neutrophil-dependent mediators of vasopermeability. *Can J Physiol Pharmacol* 1990; **68**: 836–844.
17. Varani J, Ginsburg I, Schuger L, *et al.* Endothelial cell killing by neutrophils: synergistic interaction of oxygen products and proteases. *Am J Pathol* 1989; **135**: 435–438.
18. Inauen W, Granger DN, Meininger CJ, Schelling ME, Granger HJ, Kvietys PR. Anoxia-reoxygenation-induced, neutrophil-mediated endothelial cell injury: role of elastase. *Am J Physiol* 1990; **259**: 925–931.
19. Mcdonald JA, Kelly DG. Degradation of fibronectin by human leukocyte elastase: release of biologically active fragments. *J Biol Chem* 1980; **255**: 8848–8858.
20. Twumasi DY, Liener IE. Proteases from purified sputum. Purification and properties of the elastase and chymotrypsin-like enzymes. *J Biol Chem* 1977; **252**: 1917–1926.
21. Garcia JG, Birnboim AS, Bizjos R, Del Vecchio PJ, Feuton II JG, Malik AB. Thrombin-induced increase in albumin permeability across the endothelium. *J Cell Physiol* 1986; **128**: 96–104.
22. Cooper JA, Del Vecchio PJ, Minnear FL, *et al.* Measurement of albumin permeability across endothelial monolayers *in vitro*. *J Appl Physiol* 1987; **62**: 1076–1083.
23. Weiss SJ. Tissue destruction by neutrophils. *N Engl J Med* 1989; **320**: 365–376.
24. Kawabata K, Suzuki M, Sugitani M, Imaki K, Toda M, Miyamoto T. ONO-5046: a novel inhibitor of human neutrophil elastase. *Biochem Biophys Res Comm* 1991; **177**: 814–820.
25. Johnson JL, Rajagopalan KV, Cohen HJ. Molecular basis of the biological function of molybdenum. *J Biol Chem* 1974; **249**: 859–866.
26. Ishii Y, Partridge CA, Del Vecchio PJ, Malik AB. Tumor necrosis factor-alpha-mediated decrease in glutathione increases the sensitivity of pulmonary vascular endothelial cells to $H_2O_2$. *J Clin Invest* 1992; **89**: 794–802.
27. Skutelsky E, Rudich Z, Danon D. Surface charge properties of the luminal front of blood vessel walls: an electron microscopical analysis. *Thromb Res* 1975; **7**: 623–634.
28. Baugh RJ, Travis J. Human leukocyte granule elastase: rapid isolation and characterization. *Biochemistry* 1976; **15**: 836–841.
29. Martodam RR, Baugh RJ, Twamasi DY, Liener IC. A rapid procedure for the large scale purification of elastase and cathepsin G from human sputum. *Prep Biochem* 1979; **9**: 15–31.
30. Olsson I, Venge P. Cationic proteins of human granulocytes. II. Separation of the cationic proteins of the granules of leukemic myeloid cells. *Blood* 1974; **44**: 235–246.
31. Havemann K, Gramse M. Physiology and pathophysiology of neutral proteinases of human granulocytes. *Adv Exp Med Biol* 1984; **167**: 1–20.
32. Weiss SJ, Curnutte JT, Regiani S. Neutrophil-mediated solubilization of the subendothelial matrix: oxidative and nonoxidative mechanisms of proteolysis used by normal and chronic granulomatous disease phagocytes. *J Immunol* 1986; **136**: 636–641.
33. Key NS, Platt JL, Vercellotti GM. Vascular endothelial cell proteoglycans are susceptible to cleavage by neutrophils. *Arterioscler Thromb* 1992; **12**: 836–842.
34. Partridge CA, Horvath CJ, Del Vecchio PJ, Phillips PG, Malik AB. Influence of extracellular matrix in tumor necrosis factor-induced increase in endothelial permeability. *Am J Physiol* 1992; **263**: 627–633.

ACKNOWLEDGEMENTS. The authors thank Ms Aiko Ohashi for expert technical assistance and Ms Naomi Suzuki and Ms Hiromi Kanazawa for excellent secretarial assistance.

**Received 14 July 1993; accepted 21 October 1993**

## References

[PDF_00581] Baugh R. J., Travis J. (1976). Human leukocyte granule elastase: rapid isolation and characterization.. Biochemistry.

[PDF_00507] Eiermann G. J., Dickey B. F., Thrall R. S. (1983). Polymorphonuclear leukocyte participation in acute oleic-acid-induced lung injury.. Am Rev Respir Dis.

[PDF_00522] Flick M. R., Perel A., Staub N. C. (1981). Leukocytes are required for increased lung microvascular permeability after microembolization in sheep.. Circ Res.

[PDF_00513] Fowler A. A., Hyers T. M., Fisher B. J., Bechard D. E., Centor R. M., Webster R. O. (1987). The adult respiratory distress syndrome. Cell populations and soluble mediators in the air spaces of patients at high risk.. Am Rev Respir Dis.

[PDF_00562] Garcia J. G., Siflinger-Birnboim A., Bizios R., Del Vecchio P. J., Fenton J. W., Malik A. B. (1986). Thrombin-induced increase in albumin permeability across the endothelium.. J Cell Physiol.

[PDF_00589] Havemann K., Gramse M. (1984). Physiology and pathophysiology of neutral proteinases of human granulocytes.. Adv Exp Med Biol.

[PDF_00517] Heflin A. C., Brigham K. L. (1981). Prevention by granulocyte depletion of increased vascular permeability of sheep lung following endotoxemia.. J Clin Invest.

[PDF_00575] Ishii Y., Partridge C. A., Del Vecchio P. J., Malik A. B. (1992). Tumor necrosis factor-alpha-mediated decrease in glutathione increases the sensitivity of pulmonary vascular endothelial cells to H2O2.. J Clin Invest.

[PDF_00573] Johnson J. L., Rajagopalan K. V., Cohen H. J. (1974). Molecular basis of the biological function of molybdenum. Effect of tungsten on xanthine oxidase and sulfite oxidase in the rat.. J Biol Chem.

[PDF_00570] Kawabata K., Suzuki M., Sugitani M., Imaki K., Toda M., Miyamoto T. (1991). ONO-5046, a novel inhibitor of human neutrophil elastase.. Biochem Biophys Res Commun.

[PDF_00595] Key N. S., Platt J. L., Vercellotti G. M. (1992). Vascular endothelial cell proteoglycans are susceptible to cleavage by neutrophils.. Arterioscler Thromb.

[PDF_00583] Martodam R. R., Baugh R. J., Twumasi D. Y., Liener I. E. (1979). A rapid procedure for the large scale purification of elastase and cathepsin G from human sputum.. Prep Biochem.

[PDF_00556] McDonald J. A., Kelley D. G. (1980). Degradation of fibronectin by human leukocyte elastase. Release of biologically active fragments.. J Biol Chem.

[PDF_00499] Meyrick B. (1986). Pathology of the adult respiratory distress syndrome.. Crit Care Clin.

[PDF_00529] Ohlsson K. (1980). Interactions between granulocyte proteases and protease inhibitors in the lung.. Bull Eur Physiopathol Respir.

[PDF_00586] Olsson I., Venge P. (1974). Cationic proteins of human granulocytes. II. Separation of the cationic proteins of the granules of leukemic myeloid cells.. Blood.

[PDF_00538] Plow E. F. (1982). Leukocyte elastase release during blood coagulation. A potential mechanism for activation of the alternative fibrinolytic pathway.. J Clin Invest.

[PDF_00504] Rinaldo J. E., Dauber J. H., Christman J., Rogers R. M. (1984). Neutrophil alveolitis following endotoxemia. Enhancement by previous exposure to hyperoxia.. Am Rev Respir Dis.

[PDF_00541] Rodell T. C., Cheronis J. C., Ohnemus C. L., Piermattei D. J., Repine J. E. (1987). Xanthine oxidase mediates elastase-induced injury to isolated lungs and endothelium.. J Appl Physiol (1985).

[PDF_00578] Skutelsky E., Rudich Z., Danon D. (1975). Surface charge properties of the luminal front of blood vessel walls: an electron microscopical analysis.. Thromb Res.

[PDF_00501] Taylor R. G., McCall C. E., Thrall R. S., Woodruff R. D., O'Flaherty J. T. (1985). Histopathologic features of phorbol myristate acetate-induced lung injury.. Lab Invest.

[PDF_00559] Twumasi D. Y., Liener I. E. (1977). Proteases from purulent sputum. Purification and properties of the elastase and chymotrypsin-like enzymes.. J Biol Chem.

[PDF_00525] Vedder N. B., Winn R. K., Rice C. L., Chi E. Y., Arfors K. E., Harlan J. M. (1988). A monoclonal antibody to the adherence-promoting leukocyte glycoprotein, CD18, reduces organ injury and improves survival from hemorrhagic shock and resuscitation in rabbits.. J Clin Invest.

[PDF_00510] Weiland J. E., Davis W. B., Holter J. F., Mohammed J. R., Dorinsky P. M., Gadek J. E. (1986). Lung neutrophils in the adult respiratory distress syndrome. Clinical and pathophysiologic significance.. Am Rev Respir Dis.

[PDF_00591] Weiss S. J., Curnutte J. T., Regiani S. (1986). Neutrophil-mediated solubilization of the subendothelial matrix: oxidative and nonoxidative mechanisms of proteolysis used by normal and chronic granulomatous disease phagocytes.. J Immunol.

[PDF_00568] Weiss S. J. (1989). Tissue destruction by neutrophils.. N Engl J Med.

[PDF_00531] Weitz J. I., Huang A. J., Landman S. L., Nicholson S. C., Silverstein S. C. (1987). Elastase-mediated fibrinogenolysis by chemoattractant-stimulated neutrophils occurs in the presence of physiologic concentrations of antiproteinases.. J Exp Med.

